# Programmed Interfacial Phase Separation for Spatiotemporal Fungicide Delivery via Tunable Micro/Nano Architectures

**DOI:** 10.1002/advs.77149

**Published:** 2026-08-11

**Authors:** Rui Zhang, Tongfang Jing, Leiming He, Kaidi Cui, Yuying Song, Xuewei Mao, Lidong Cao, Lin Zhou

**Affiliations:** ^1^ College of Plant Protection Henan Agricultural University Zhengzhou Henan People's of Republic China; ^2^ Henan Key Laboratory of Creation and Application of New Pesticide Henan Agricultural University Zhengzhou Henan People's of Republic China; ^3^ State Key Laboratory of High‐Efficiency Production of Wheat‐Maize Double Cropping Zhengzhou Henan People's of Republic China; ^4^ Institute of Plant Protection Chinese Academy of Agricultural Sciences Beijing People's of Republic China

**Keywords:** liquid–liquid phase separation, metal‐polyphenol network, precision agriculture, self‐assembly

## Abstract

Translating dynamic liquid‐liquid phase separation into robust solid‐state architectures remains a challenge in materials engineering. Here, we report a composition‐driven interfacial assembly strategy that couples supramolecular coacervation with metal‐phenolic coordination to engineer tunable fungicide carriers. By modulating the mass ratio between tea polyphenols (TP) and non‐ionic surfactants, we generate fluid templates that are kinetically trapped via pH‐triggered iron complexation. This one‐pot protocol allows for the precise regulation of the micro‐to‐nano population ratio, effectively addressing the dimensional mismatch between soil retention and systemic uptake. The resulting architecture achieves a functional division: microcapsules function as stationary reservoirs for rhizosphere protection, while nanocapsules act as mobile vectors for systemic curative action. Furthermore, the metal‐phenolic shell exhibits pathogen‐responsive disassembly upon exposure to fungal virulence factors, including oxalic acid and cellulases secreted by *Fusarium* pathogens. Validated in a peanut root rot model, this system demonstrates improved spatiotemporal efficacy and reduced aquatic toxicity toward zebrafish compared to commercial formulations. Consequently, this work presents a versatile methodology for structuring dynamic liquid interfaces based on TP‐surfactant supramolecular interactions, offering a potent solution for precision agriculture through the controlled solidification of supramolecular assemblies.

## 1 Introduction

Soil‐borne fungal pathogens, such as *Fusarium solani* and *Fusarium oxysporum*, pose a severe threat to agricultural sustainability [1]. However, effective management is difficult because fungicide efficacy is often compromised by adsorption onto the heterogeneous soil matrix and degradation by dynamic microbial communities in the rhizosphere [2]. To address these threats, researchers have developed various agrochemical strategies. Conventional approaches usually focus on microencapsulation technologies that employ in situ polymerization or complex coacervation to build rigid shells like polyurea and polyurethane [3, 4]. These microscale barriers protect the payload from UV radiation and microbial degradation and minimize soil adsorption while acting as stationary reservoirs within the rhizosphere for preventative protection [5, 6]. In contrast, the demand for curative action has driven the development of nanotechnology‐based delivery systems including mesoporous silica nanoparticles and metal–organic frameworks (MOFs) [7, 8]. These nanoscale vectors are often functionalized with specific amino acids or carbohydrates to navigate symplastic pathways and are necessary for overcoming the rigid plant cell wall to achieve systemic uptake and translocation [9, 10, 11]. Nevertheless, a fundamental dimensional mismatch persists: microcapsules are too large to penetrate plant tissues for curative effects, while nanocarriers are prone to rapid leaching and fail to provide long‐term soil retention [12, 13]. Current multi‐scale formulations are typically prepared by physically blending separately synthesized micro‐ and nanoparticles, a process that requires distinct kinetic regimes, reaction conditions, and independent synthesis and purification steps [5]. This disjointed and fragmented fabrication strategy often results in compositional heterogeneity, batch‐to‐batch variability, and limited structural integration [14, 15, 16], while increasing formulation complexity and failing to address the conflicting spatiotemporal requirements of comprehensive crop protection [17].

Beyond these structural limitations, the imperative for sustainable agriculture has propelled natural biomass‐derived materials as compelling alternatives to synthetic polymers, owing to their intrinsic biocompatibility and biodegradability [18]. Natural polyphenols, such as tea polyphenols (TP) and tannins, have attracted attention for their intrinsic antioxidant and antimicrobial properties, as well as their versatile adhesive chemistry and supramolecular assembly potential [19, 20]. Synthetic polymers often require complicated design processes and costly functionalization to confer bio‐responsiveness. In comparison, polyphenols can spontaneously undergo coordination with metal ions to form metal‐phenolic networks [21]. These networks provide a green and modular platform for surface engineering that responds inherently to environmental cues like pH changes and competitive chelators. This approach parallels functionalization strategies in advanced biomedical hydrogels where natural small molecules enhance biocompatibility and bioactivity [22]. Utilizing the supramolecular interactions of natural polyphenols offers a pathway to bridge the gap between ecological safety and therapeutic efficacy, replacing inert synthetic shells with dynamic, pathogen‐responsive architectures.

Translating the supramolecular potential of polyphenols into the desired multiscale architecture requires a dynamic assembly mechanism beyond simple coordination chemistry. Bioinspired liquid‐liquid phase separation (LLPS) offers a promising approach [23]. In biology this thermodynamic process organizes complex biochemical reactions by creating membraneless organelles with distinct boundaries [24, 25]. Applying this principle to agriculture provides a pathway to generate discrete carriers from a continuous liquid phase. However, a critical gap remains in controlling this process to produce specific sizes. Microcapsule synthesis typically relies on emulsion templating to stabilize large droplets whereas nanocarrier fabrication predominantly exploits rapid precipitation kinetics [26, 27]. Integrating these divergent regimes within a single system is difficult. A potential solution may lie in the intrinsic chemical properties of standard agrochemical components. Non‐ionic surfactants are ubiquitous in pesticide formulations and possess hydrophilic segments that theoretically offer binding sites for polyphenols. However, the specific interfacial assembly behavior between these agents and natural polyphenols has not been fully explored. If such supramolecular interactions could be harnessed to induce a composition‐driven phase separation they might provide a mechanism to regulate carrier dimensions programmatically. Validating this hypothesis would establish a unified synthetic framework to enable a synergistic functional distribution between prevention and cure.

Herein, we validate this concept by establishing a generalized platform for programmed spatiotemporal fungicide delivery via interfacially induced phase separation. Our investigations reveal that the affinity between hydrophilic polyether segments and phenolic hydroxyl moieties acts as a generic driving force for interfacial accumulation across a spectrum of non‐ionic surfactants. Employing styrene phenol polyoxyethylene ether (SPPE) as a representative model, we confirm that cooperative hydrogen bonding establishes a concentrated supramolecular template that spontaneously segregates into a polyphenol‐dense coacervate phase. To solidify this transient liquid template, we employ an interfacial titration strategy inspired by metal‐polyphenol network‐encapsulated coacervates, which integrates a phase‐transition switch with dynamic kinetic trapping [28, 29]. The fluid coacervate is initially maintained in an acidic environment containing ferric ions. Subsequent pH modulation triggers rapid ligand deprotonation and drives the formation of stable tris‐catecholate‐iron complexes that kinetically lock the template structure [30, 31].

**SCHEME 1:**
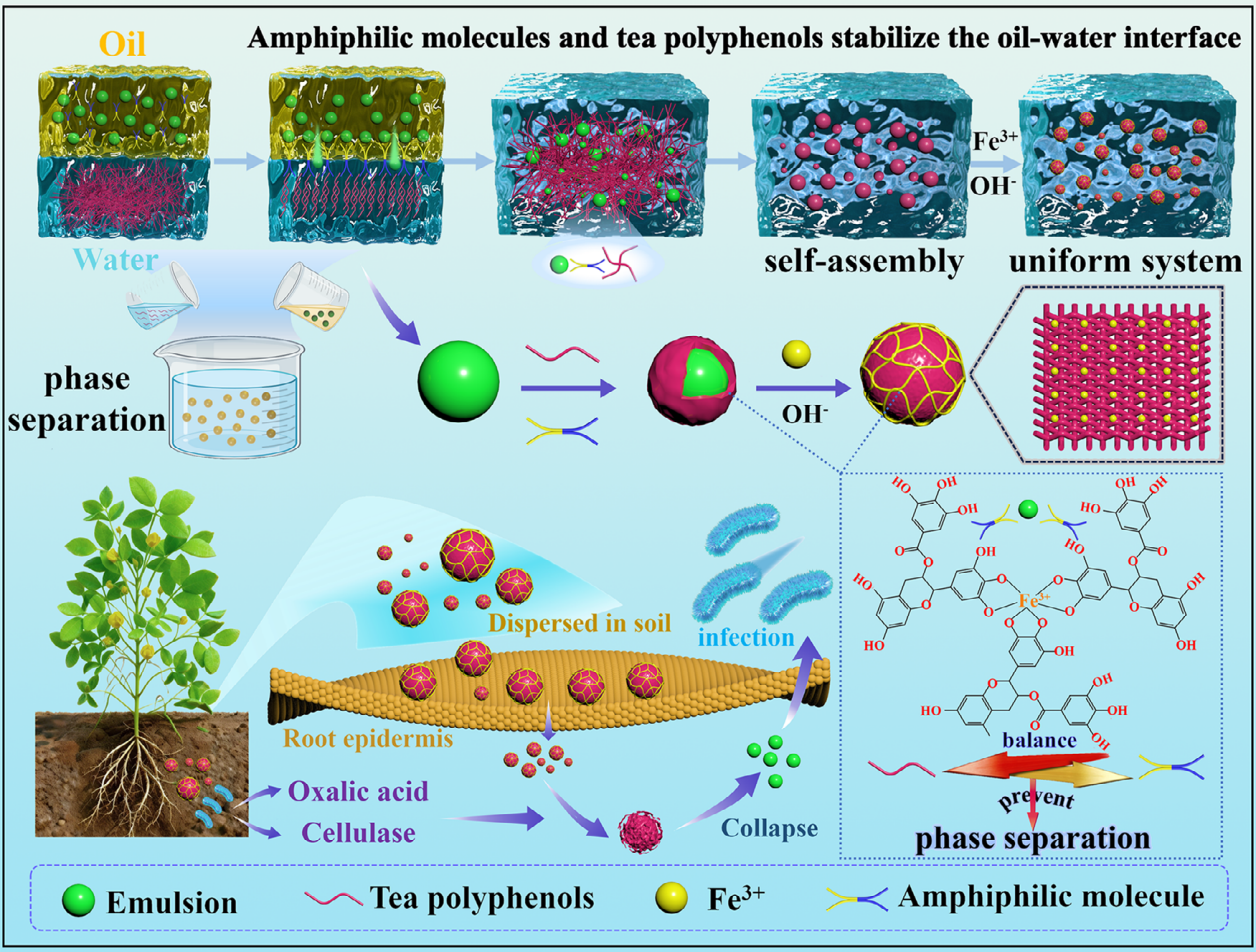
Programming Spatiotemporal Fungicide Delivery. Schematic illustration of the surfactant‐mediated interfacial phase separation strategy for engineering composition‐tunable TP@Fe multiscale vectors (NMCs). The system features a pathogen‐instructed gating mechanism that triggers disassembly in response to the specific virulence microenvironment of *F. solani* and *F. oxysporum*.

This one‐pot protocol allows for the engineering of tunable nano/micro capsules (NMCs) under ambient conditions, where the nanocapsule fraction can be precisely regulated from 54% to 96% simply by adjusting the precursor stoichiometry. This capability effectively overcomes the key limitations of traditional blending, including its fragmented multi‐step fabrication process, poor structural integration between micro‐ and nanoscale components, and limited compositional tunability. The resulting multiscale architecture achieves a synergistic division of labor where microcapsules function as a stationary rhizosphere reservoir for long‐term prevention and nanocapsules act as mobile vectors for systemic curative action. Furthermore, the incorporated iron centers create a pathogen‐instructed gating mechanism. Unlike passive release systems this network disassembles specifically in response to fungal virulence factors including the secretion of oxalic acid and cellulases [32]. This mechanism converts the attack signal of the pathogen into a trigger for fungicide release. Validated in a peanut root rot model this system exhibits improved spatiotemporal efficacy and enhanced environmental biosafety compared to commercial formulations (Scheme 1). This work establishes a versatile strategy for engineering intelligent agricultural materials through the controlled solidification of dynamic liquid interfaces and offers a solution to the dimensional and ecological challenges of modern crop protection.

## 2 Results and Discussion

### 2.1 Mechanism of Composition‐Driven Coacervation and Kinetic Trapping

**FIGURE 1:**
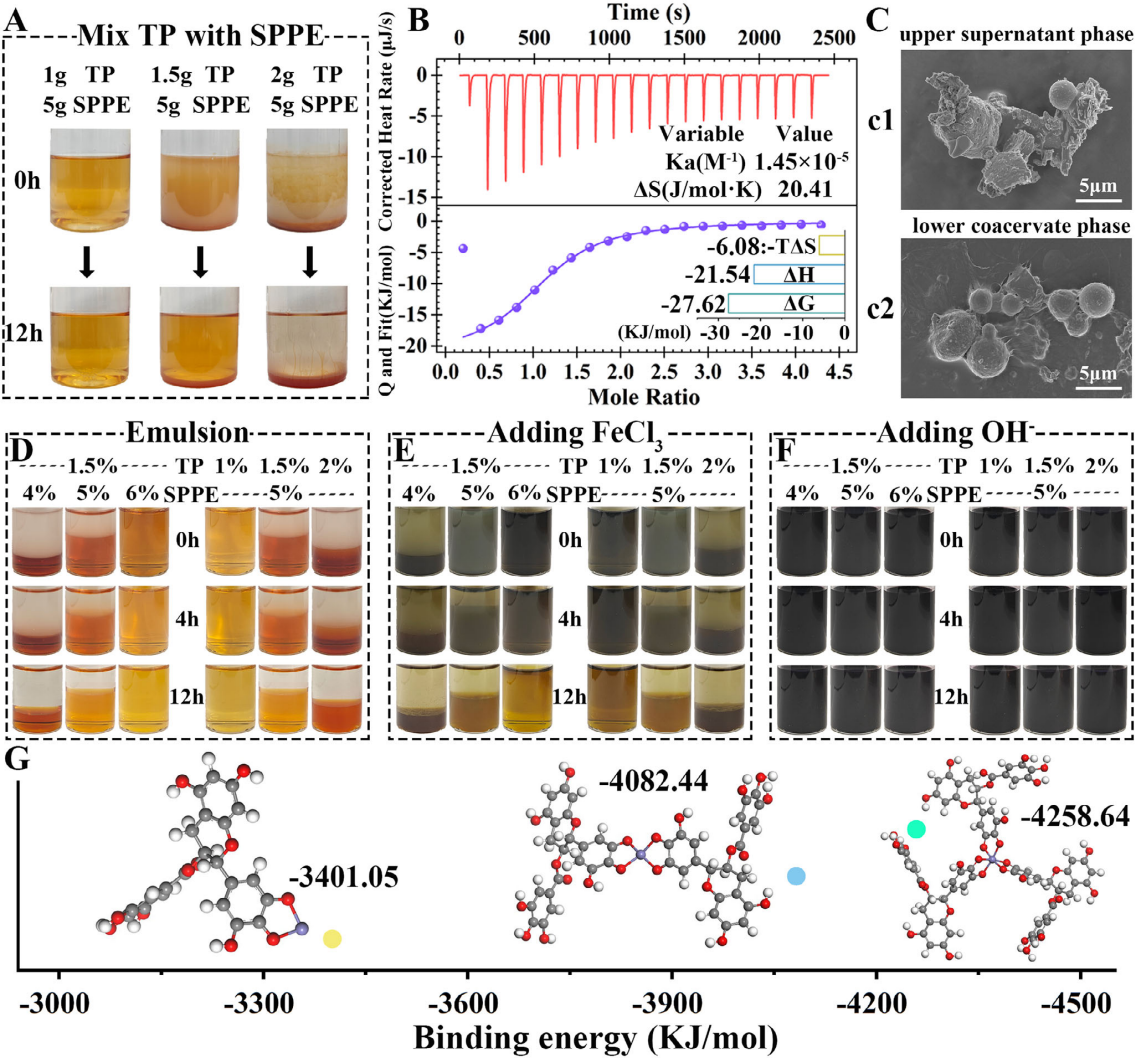
Mechanism of composition‐driven interfacial coacervation and kinetic trapping. (A) The phase evolution of binary aqueous mixtures of SPPE with increasing TP concentrations. (B) Thermodynamic binding profiles of the SPPE‐TP interaction. (C) SEM micrograph of the dried residue from the upper supernatant phase (c1) and the lower coacervate phase (c2) after phase separation of the oil‐water biphasic emulsion formed by SPPE and TP. (D–F) The phase evolution of oil‐water biphasic emulsion mixtures of SPPE with increasing TP concentrations. (D) Time‐dependent macroscopic observations (0‐12 h) of emulsions with varying TP ratios. (E) Macroscopic evolution of the emulsion after introducing Fe^3+^ (acidic condition) over 12 h. Note the distinct stratification into a clear supernatant and a dark sediment. (F) Macroscopic observation of the system following OH^−^‐triggered pH modulation over 12 h. (G) DFT‐calculated binding energy landscapes for the coordination of Fe^3+^ with TP.

The phase behavior of the binary aqueous precursors was first investigated using SPPE as the representative model to validate the hypothesis of a composition‐driven assembly mechanism. As shown in Figure 1A, the interaction is controlled by the mass ratio. At a TP to SPPE ratio of 1:5, the mixture remains a homogeneous, transparent solution. However, increasing the mass ratio (TP/SPPE) to 1.5:5 induces a phase transition where the system becomes turbid and gradually forms an orange‐red coacervate phase. Further elevating the polyphenol proportion to 2:5 results in an increase in the volume of the coacervate phase. This concentration‐dependent evolution indicates that the assembly is driven by specific intermolecular associations rather than random physical mixing.

To assess the generality of this composition‐driven assembly, the investigation was extended to other non‐ionic surfactants (Figure S1). The screening included structurally diverse candidates such as NS‐500LQ, EL‐40, OP‐10, Span‐80, Tween‐80, and AEO‐9. With the exception of AEO‐9, all selected surfactants displayed consistent phase behavior, in which increasing the TP concentration triggered a transition from a homogeneous solution to a turbid phase‐separated system. This applicability across varying molecular architectures, including sorbitan derivatives and differing polyoxyethylene chain lengths, demonstrates the stability of the supramolecular interaction. The deviation observed with AEO‐9 is attributed to its flexible linear aliphatic architecture, which contrasts with the aromatic or cyclic hydrophobic cores present in the successful candidates. This suggests that steric rigidity or specific spatial topology is necessary to stabilize the supramolecular network against solvation and facilitate macroscopic phase separation. These results indicate that the proposed strategy is applicable to non‐ionic surfactants possessing aromatic or cyclic hydrophobic cores.

Having established the bulk phase behavior, the thermodynamic origin of the specific association between SPPE and TP was quantified via Isothermal Titration Calorimetry (ITC) (Figure 1B). An enthalpy change of −21.54 kJ/mol was recorded, indicating an exothermic binding profile. This value falls within the typical range of hydrogen bonding [33]. This composition‐dependent behavior is also observed at the oil‐water interface (Figure 1D). In this system, an aqueous TP phase was introduced into an oil phase containing cyclohexanone, pyraclostrobin, and SPPE. Consistent with the bulk aqueous observations, the interface remains homogeneous and transparent at TP to SPPE ratios of 1:4 and 1:5. However, increasing the mass ratio (TP/SPPE) to 1.5:5 or higher triggers phase separation. Additional investigations confirmed that this interfacial assembly capability applies to other systems, as surfactants, including NS‐500LQ, EL‐40, OP‐10, Span‐80, and Tween‐80, also exhibited characteristic phase separation behaviors upon interfacial contact (Figures S2 and S3). Scanning Electron Microscopy (SEM) analysis shows a heterogeneous morphology where nascent capsule‐like structures are embedded within an amorphous gel network, with a noticeably higher abundance of capsule‐like structures observed in the coacervate phase than in the supernatant phase (Figure 1C, Figure S4). This observation confirms that the initial shell exists as a dynamic liquid‐liquid phase‐separated layer lacking discrete structural integrity, while also suggesting that the coacervate phase may serve as the primary source for subsequent capsule formation.

The solidification of this fluid interfacial layer is achieved through the interfacial titration strategy. The introduction of Fe^3+^ initiates the first stage of the transition (Figure 1E). An immediate color change to dark green indicates the coordination of metal centers with phenolic motifs. Taking the 2:5 (TP:SPPE) ratio as a representative case, the system exhibits distinct stratification after 0 to 12 h characterized by a transparent supernatant overlying a dense and dark coacervate phase. Notably, this bottom layer retains a fluid consistency which distinguishes it from solid precipitates. This macroscopic observation confirms that SPPE‐mediated hydrogen bonding drives the efficient interfacial enrichment of TP. Consequently, the resulting Fe^3+^‐TP complexes are preferentially sequestered within this dynamic liquid interface. SEM and Transmission Electron Microscopy (TEM) observations of systems prepared at varying SPPE/TP ratios revealed the coexistence of both amorphous capsule‐like structures and spherical nanocapsules in both the supernatant and coacervate phases (Figures S5 and S6), indicating that the system resides in an incipient, kinetically arrested metastable state rather than a thermodynamically equilibrated structure. Notably, compared with the supernatant phase, the coacervate phase exhibited a higher proportion of nanocapsular structures, suggesting that a coacervate‐rich environment favors further densification and interfacial reorganization [34]. In this regard, the coacervate phase may serve as the primary domain for nanocomponent maturation, where enhanced local coordination interactions and elevated packing density synergistically drive the structural evolution of nanoscale assemblies [35]. Microstructural characterization further confirms the distinct evolutionary origins of the micro‐ and nanoscale components.

However, the acidic environment of the Fe^3+^ source maintains a low coordination valency [30]. This characteristic preserves the fluid nature of the interface. The solidification step is triggered by pH modulation as presented (Figure 1F). The addition of sodium hydroxide deprotonates the phenolic ligands. This process shifts the thermodynamic equilibrium toward the formation of stable tris‐catecholate‐iron complexes. To validate the driving force behind this solidification, molecular simulations were employed to calculate the binding energies between Fe^3+^ and TP ligands (Figure 1G). The calculations reveal binding energies of −3401.05, −4082.44, and −4258.64 kJ/mol for the coordination of one, two, and three polyphenol molecules, respectively. The energy gain associated with the tris‐coordinated state confirms the formation of a stable network, which locks the transient liquid template into a solid structure. SEM and TEM images further confirm the formation of robust capsule structures (Figures S5 and S6). Consequently, the transient template is kinetically trapped into a rigid solid‐state network, resulting in the rapid formation of a homogeneous dispersion.

### 2.2 Molecular Dynamics Insights Into Composition‐Dependent Interfacial Assembly

**FIGURE 2:**
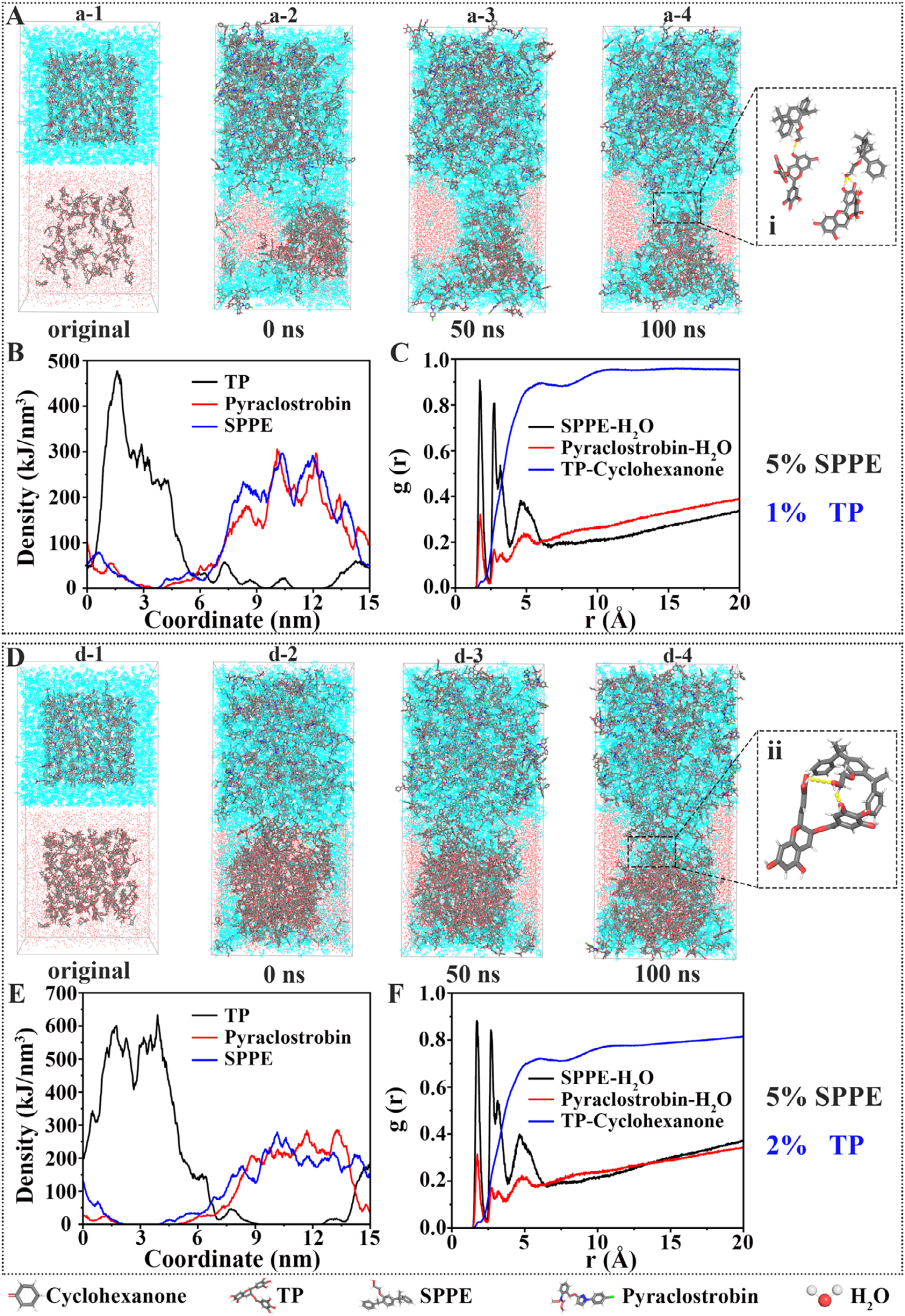
Molecular dynamics insights into composition‐dependent interfacial assembly. (A–C) Simulation results for the dilute system (1% TP). (A) Representative equilibrium snapshots. The magnified view (i) shows the local molecular configuration. (B) Z‐axis density profiles showing limited interfacial accumulation of TP. (C) RDF highlighting the intact hydration shell around surfactant chains. (D–F) Simulation results for the concentrated system (2% TP). (D) Representative snapshots showing a condensed interfacial layer. The magnified view (inset ii) visualizes the supramolecular bridging mechanism where TP crosslinks surfactant chains via hydrogen bonding. (E) Density profiles revealing a sharp increase in TP concentration at the interface. (F) RDF confirming the persistence of hydration within the coacervate network.

To understand the microscopic mechanism of the observed phase behavior, all‐atom molecular dynamics (MD) simulations were conducted to map the interfacial assembly evolution. At the lower TP concentration of 1%, the interface exhibits a loosely organized assembly (Figure 2A, Figure S9). Although hydrogen bonding drives polyphenol migration to the surfactant layer, the density profile indicates that the interfacial accumulation remains limited (Figure 2B). Under these conditions, the hydrophilic polyether segments of the surfactants retain intact hydration shells, thereby preserving effective steric stabilization [36]. This hypothesis is quantitatively corroborated by the radial distribution function (RDF) analysis (Figure 2C). The distinct sharp peak observed in the surfactant‐water pair confirms the presence of a dense hydration layer surrounding the polyether chains, which is critical for maintaining the emulsion's stability. Consequently, the population of uncomplexed surfactants retains intrinsic interfacial activity, yielding the macroscopically homogeneous dispersion observed experimentally.

In contrast, increasing the TP concentration to 2% causes a transition toward a condensed state (Figure 2D, Figure S10). The simulation demonstrates a saturation phenomenon where TP occupy the hydrophilic polyether segments of the SPPE. The magnified view in Figure 2D‐ii unveils a bridging mechanism wherein polyphenols act as supramolecular crosslinkers. They crosslink adjacent surfactant molecules via an extensive hydrogen bond network involving the phenolic hydroxyls and the oxygen‐containing hydrophilic motifs of the surfactants. This binding displaces the hydration water molecules and reduces the original steric repulsion between surfactant chains. By disrupting the emulsifying capability of the free surfactants, this bridging effect destabilizes the emulsion and induces the formation of a coacervate phase [37]. The structural transformation is supported by the density profiles (Figure 2E, Figure S11), where the polyphenol density at the interface shows a sharp increase, indicating the formation of a condensed phase. The RDF reveals that the surfactant‐water interaction peak remains prominent and comparable to the dilute system (Figure 2C,F). This persistence of hydration indicates that the coacervate phase retains a significant solvent content. This confirms that the interface forms a hydrated coacervate rather than a solid precipitate. Such a dynamic liquid state is essential for the subsequent kinetic trapping. These simulation results provide structural evidence that the interfacial assembly is governed by a specific stoichiometry, where the polyphenol concentration determines the connectivity and density of the supramolecular network [38, 39].

### 2.3 Morphological Regulation and Structural Characterization

**FIGURE 3:**
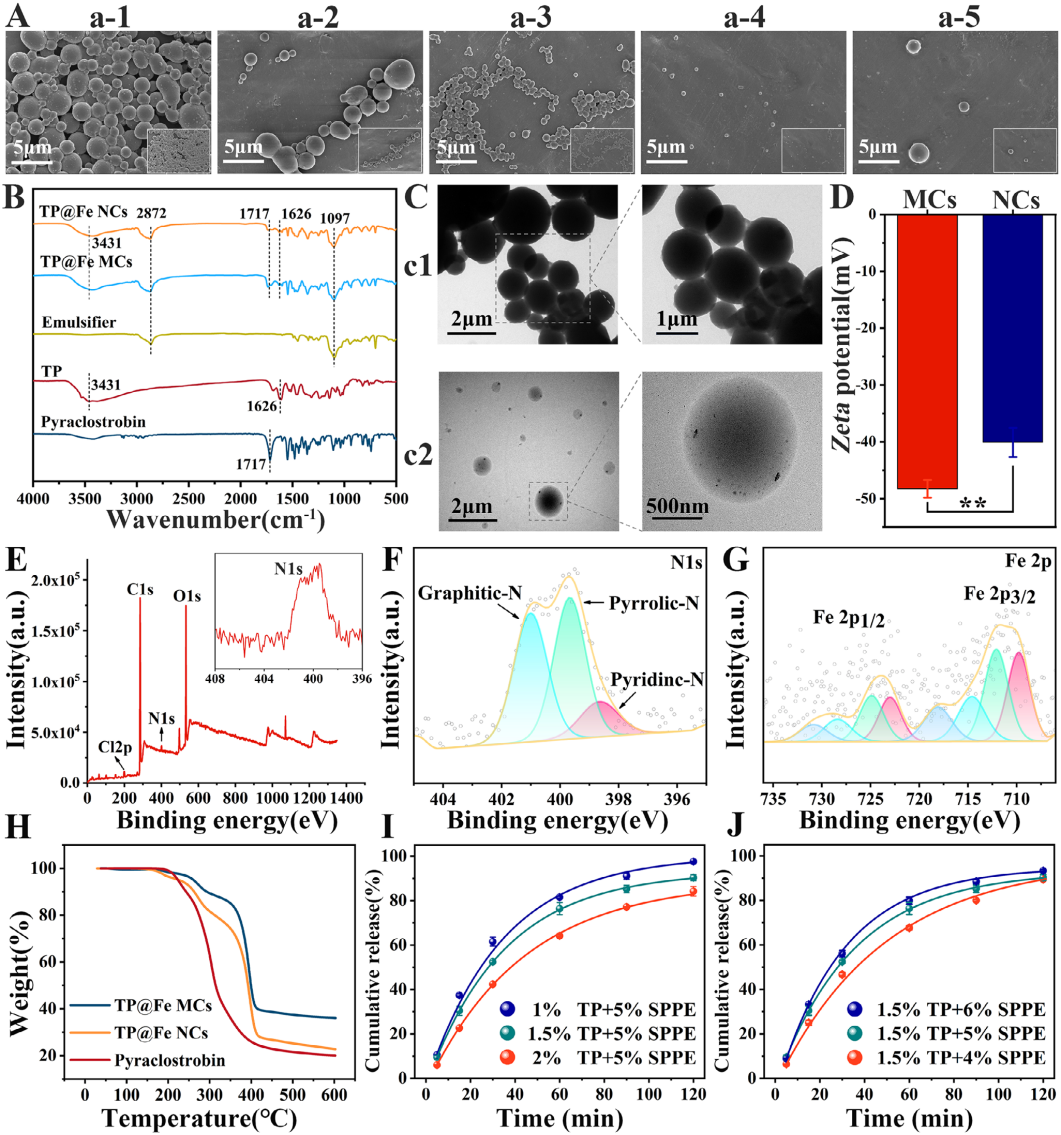
Morphological regulation and structural characterization of the TP@Fe multiscale architectures. (A) SEM images illustrating the stoichiometry‐driven morphological evolution of the NMCs. Panels (a‐1) to (a‐5) correspond to formulations with varying nanocapsule:microcapsule ratios (from 58:42 to 95:5). (B) FTIR spectra. (C) TEM micrographs of the NMCs. (D) *Zeta* potential measurements. (E–G) Surface elemental analysis via XPS. (H) TGA curves. (I, J) Pyraclostrobin release profiles exhibiting tunable kinetics dependent on the (I) TP concentration and (J) SPPE ratio.

Guided by the molecular simulations highlighting the stoichiometry‐dependent networking, we investigated how precursor ratios dictate the final particle morphology. SEM images show that microcapsules and nanocapsules coexist across all formulations (Figure 3A, Table S2). However, adjusting the precursor ratio alters the proportion of these two populations. At high TP concentrations, a larger number of microcapsules appear alongside the nanocarriers. As the TP to SPPE ratio decreases, the proportion of microcapsules decreases. This morphological variation is controlled by the interaction between surfactant activity and interfacial network density [40]. At higher TP ratios, the extensive hydrogen bonding creates a dense interfacial film [41]. This robust scaffold kinetically traps larger microdroplets, preventing their fragmentation into smaller units during emulsification. Conversely, lowering the TP concentration increases the availability of free surfactant molecules at the interface. The higher surfactant concentration reduces the interfacial tension, which facilitates the generation of finer droplets. Furthermore, the reduced crosslinking density maintains a more fluid interface, allowing the system to evolve toward a nanoscale dispersion. Collectively, these results confirm that the TP density acts as a pivotal regulator of capsule dimensions.

To analyze the chemical structure of the components, Fourier Transform Infrared Spectroscopy (FTIR) was performed (Figure 3B). The spectrum retains the C═O stretching vibration at 1717 cm^−^^1^, which originates from the pyraclostrobin structure. Concurrently, the characteristic peaks at 3431 and 1626 cm^−^^1^ are attributed to the tea polyphenols, corresponding to the phenolic hydroxyl stretching and the aromatic skeletal vibration (C═C), respectively. The spectrum retains the characteristic peaks of SPPE at 2872 and 1097 cm^−^^1^, which are attributed to the aliphatic C─H stretching and the C─O─C ether stretching of the hydrophilic polyoxyethylene segments. These spectral features confirm the successful co‐assembly of the components. Following this chemical analysis, the colloidal properties were determined via *Zeta* potential measurements (Figure 3D). Both microcapsules and nanocapsules exhibit negative values ranging from −40 to −50 mV. This electrostatic repulsion, resulting from the deprotonation of surface phenolic groups, contributes to the stability of the aqueous dispersion.

Transmission Electron Microscopy (TEM) images show the internal architecture of the fractionated populations (Figure 3C), displaying a core–shell structure where a dense layer encapsulates the inner material. High‐resolution imaging of the nanocapsules confirms a uniform spherical morphology. Surface elemental states were analyzed through X‐ray Photoelectron Spectroscopy (XPS) (Figure 3E–G, Figure S7). The survey spectrum identifies C, O, N, Cl, and Fe as the constituent elements. The detection of the Cl 2p signal confirms the encapsulation of the pyraclostrobin cargo. Furthermore, the Fe 2p spectrum displays major peaks at 711.2 and 724.8 eV. These binding energies are characteristic of trivalent iron centers coordinated with oxygen ligands in a metal‐organic network.

Thermogravimetric analysis (TGA) evaluates the thermal stability of the supramolecular shell (Figure 3H, Figure S12). Compared to free pyraclostrobin, the encapsulated formulation displays a delayed decomposition. This stability is attributed to the rigid metal‐phenolic network which acts as a thermal barrier. In addition to thermal stability, the carrier allows for tunable release kinetics (Figure 3I,J, Figures S13 and S14). Increasing the TP to surfactant mass ratio slows the release rate. This trend is consistent with the morphological changes where higher polyphenol concentrations generate a higher fraction of microcapsules. These microcapsules create stronger diffusion barriers resulting in sustained release.

### 2.4 Tunable Fractionation and Antifungal Efficacy

**FIGURE 4:**
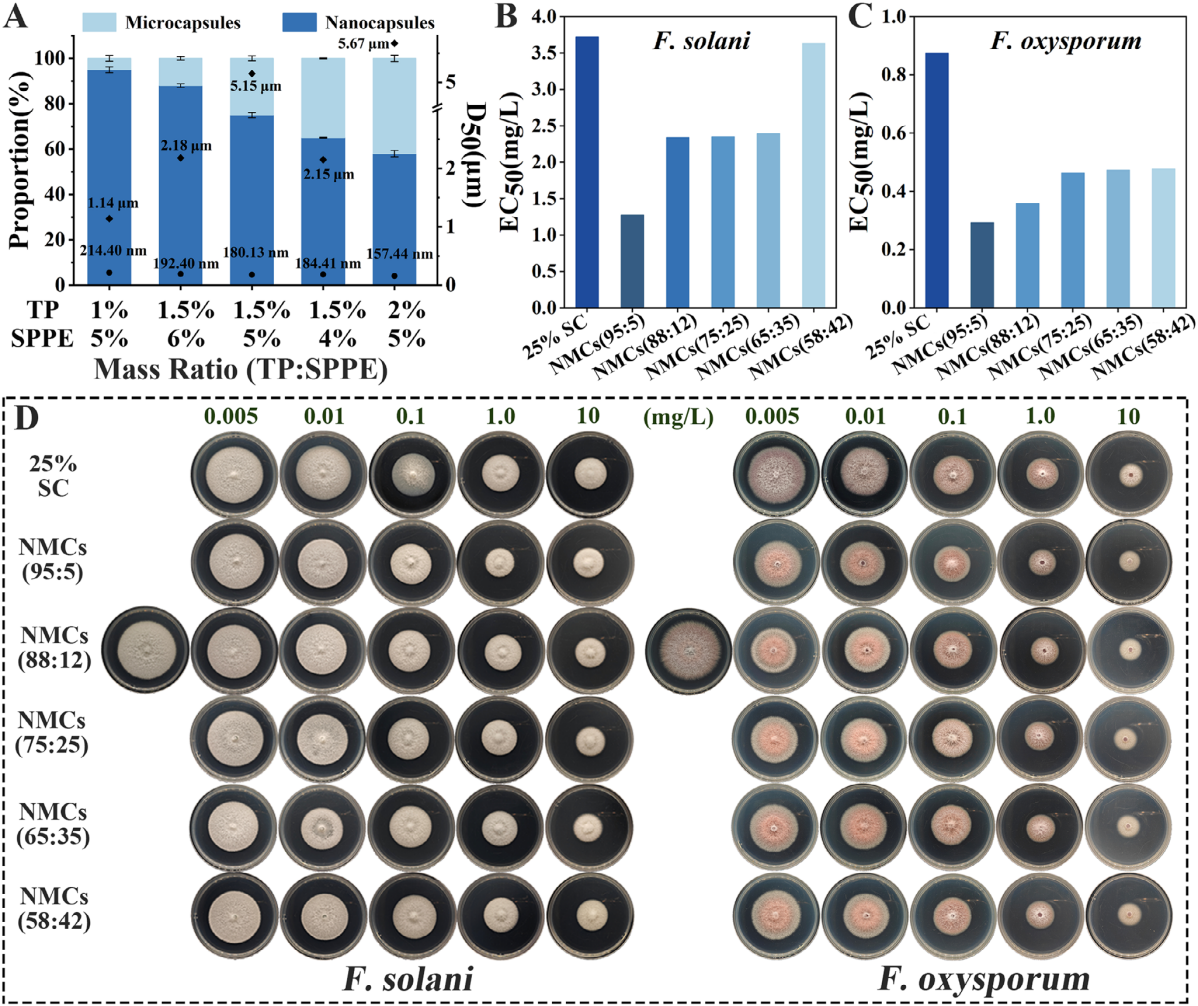
Tunable population distribution and size‐dependent in vitro antifungal efficacy. (A) Modulation of the microcapsule (MC) and nanocapsule (NC) proportions and average diameter governed by the precursor concentrations of TP and SPPE. (B, C) The EC_50_ values against (B) *F. solani* and (C) *F. oxysporum*. (D) Representative photographs of mycelial growth inhibition on agar plates.

To analyze the population distribution, the particle size was mapped against the precursor mass ratio (Figure 4A). This method allows for control over the composition based on the TP to SPPE ratio (Table S2). Specifically, the system changes from a primarily nanoscale state at low TP concentrations to a mixed state, where the nanocapsule fraction decreases to 58% as the microcapsule population expands. Compared with previously reported hierarchical delivery systems, which typically require stepwise fabrication (e.g., nanocapsule synthesis followed by secondary assembly such as spray drying) [42], such approaches further suffer from restricted structural programmability and compositional tunability. In contrast, our composition‐driven interfacial assembly enables direct one‐pot formation of hybrid capsules, with a continuously tunable nanocapsule fraction ranging from 54% to 96%. Beyond modulating the fractional distribution the precursor stoichiometry critically dictates the geometric dimensions of both carrier populations. Increasing the tea polyphenol concentration induces a divergent evolution in particle size. The hydrodynamic diameter of the nanocapsules consistently decreases from 214.40 to 157.44 nm which indicates that a higher crosslinking density leads to a more compact supramolecular packing. Conversely the microcapsules exhibit a significant expansion in average diameter from 1.14 to 5.67 µm. This phenomenon arises because the rapid interfacial rigidification acts as a mechanical barrier that arrests droplet fragmentation to yield larger microcapsules whereas the enhanced crosslinking density concurrently constricts the nanocapsules into more compact structures [43].

In vitro antifungal assays against *F. solani* and *F. oxysporum* compared the bioactivity of prepared NMCs with a commercial 25% suspension concentrate (SC) (Figure 4B,C). The bioactivity shows a size‐dependent correlation, where potency increases with the nanocapsule fraction. Consequently, the NMCs‐95:5 formulation, which contains the highest nanocapsule fraction, yielded the lowest EC_50_ values for both pathogens. Mycelial growth assays support this trend showing the highest inhibition by formulations with high nanoscale content (Figure 4D). This increased efficacy results from the larger specific surface area of the nanocapsules, which improves contact and bioavailability at the fungal hyphae [44].

### 2.5 Control Efficacy and Translocation Mechanism

**FIGURE 5:**
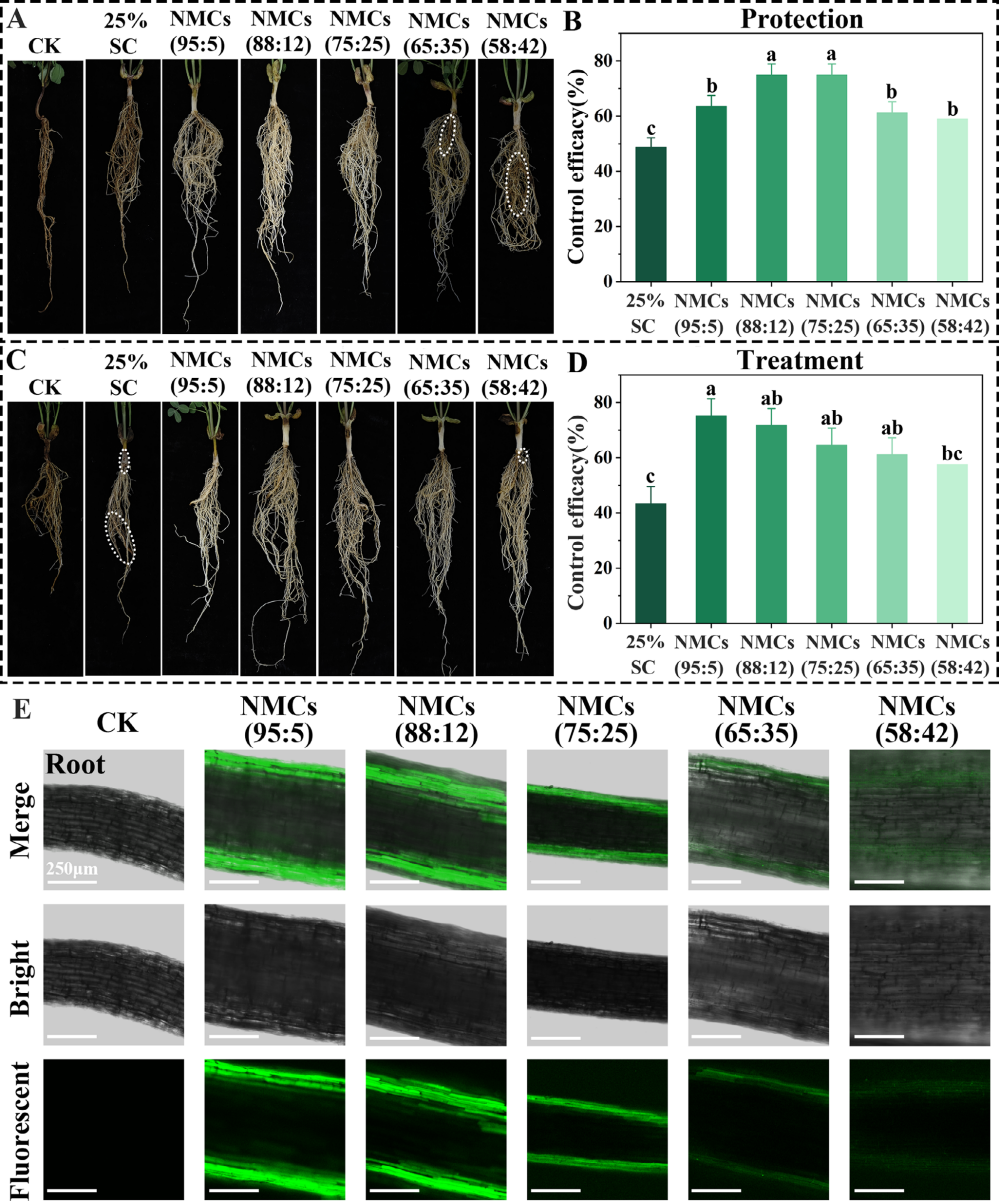
Spatiotemporal disease control efficacy and size‐dependent translocation mechanism in vivo. (A, B) Preventative assessment: (A) Representative photographs and (B) control efficacy rates of peanut plants treated with pyraclostrobin formulations (100 mg/L) prior to *F. oxysporum* infection. (C, D) Curative assessment: (C) Morphology of plants and (D) corresponding control efficacy rates treated after infection establishment. (E) CLSM images of root segments tracking the internal distribution of fluorescently labeled NMCs.

To validate the spatiotemporal functional division in vivo, pot experiments were designed to decouple the preventative and curative contributions of the respective carrier populations. Under the preventative protocol, untreated controls exhibited infection symptoms characterized by root necrosis and blackening (Figure 5A). While the commercial SC provided limited mitigation, the engineered formulations demonstrated improved protection influenced by the micro‐to‐nano mass ratio. The control efficacy analysis identifies that the NMCs‐88:12 and NMCs‐75:25 formulations showed the highest efficacy as presented (Figure 5B). The data indicates a functional trade‐off. The reduced performance of nano‐dominant variants may be attributed to insufficient long‐term soil retention. Conversely, the limitation of micro‐rich systems could stem from restricted systemic uptake capabilities [45]. These results support the need for a balanced multiscale architecture for effective preventative soil management.

In addition, the curative potential was assessed using a therapeutic protocol designed to test the systemic translocation capability of the carriers. The untreated plants exhibited tissue necrosis extending from roots to stems (Figure 5C). While the commercial SC group exhibited partial mitigation with persistent tissue discoloration, the capsule‐treated groups largely maintained a healthy morphology. The exception was the microcapsule‐rich NMCs‐58:42 group which displayed necrotic lesions in localized root zones. In terms of control efficacy, the commercial formulation provided the lowest protection rate of approximately 40% whereas all encapsulated systems demonstrated higher curative action (Figure 5D). Unlike the preventative scenario, curative efficacy exhibited a negative correlation with microcapsule content. The potency peaked in the nano‐dominant NMCs‐95:5 group and declined as the formulation shifted toward the micro‐rich NMCs‐58:42 ratio. This trend may indicate the higher biological mobility of the nanoscale fraction. The nano‐dominant carriers effectively penetrate physical barriers to target internal pathogens and function as mobile vectors for systemic therapy [7]. In contrast, the decline in efficacy in micro‐rich formulations highlights a dimensional constraint where larger microcapsules are retained at the rhizoplane and fail to penetrate internal tissues to neutralize established infections [6].

To rigorously evaluate curative potential against preestablished infections, we confirmed baseline fungal invasion at 7 days post germination via visual root necrosis and qPCR (Figure S15). After treatment, untreated controls exhibited progressive necrosis (Figure 5C). While the commercial SC group provided only partial mitigation, plants treated with capsules maintained a healthy morphology. Molecular quantification after the 15 day treatment further confirmed this efficacy (Figure S16, Table S3). Compared to the high fungal biomass in infected controls, fungicide treatments significantly reduced pathogen colonization. Notably, *Fusarium* signals in the nano dominant formulations dropped near the detection threshold. These results verify that mobile nanocapsules successfully penetrate root tissues to eradicate internal pathogens.

To visualize size‐dependent translocation, confocal laser scanning microscopy (CLSM) tracked the spatial distribution of fluorescently labeled carriers within the plant vasculature (Figure 5E). Roots treated with the nano‐dominant NMCs‐95:5 formulation displayed fluorescence signals within the central vascular cylinder. This confirms radial transport from the epidermis to the xylem. Conversely, the internal fluorescence intensity decreased as the microcapsule fraction increased toward the NMCs‐58:42 ratio. This size‐exclusion effect was also observed in the stem tissues (Figure S17). Only the NMCs‐95:5 group exhibited a continuous fluorescent pathway whereas micro‐rich formulations yielded sporadic signals. These microscopic observations align well with the macroscopic efficacy data. The strong internal fluorescence indicates that nanocapsules successfully traverse cell wall barriers to reach vascular tissues where fungal infections reside. This mobility likely explains their higher curative control rates. Conversely, the weak internal fluorescence of formulations with higher microcapsule content suggests these larger particles remain outside the root. These images visually support the proposed functional division. The nanocapsules act as systemic vectors for internal therapy, while the microcapsules serve as stationary reservoirs for soil prevention. Such size‐dependent localization and functional partitioning result in spatially differentiated delivery, thereby enabling spatially resolved disease control across multiple infection niches.

These in vivo results highlight the distinct advantages of our dual functional system. Conventional microsystems offer long term soil protection but cannot penetrate tissues for curative action. While nanoscale carriers enable internal therapy, they suffer from rapid environmental leaching. Physical blending of these scales often causes instability and phase separation. Our single step LLPS architecture overcomes these limitations by stably integrating both functionalities. The microcapsules ensure prolonged rhizosphere retention for prevention, while the mobile nanovectors provide rapid pathogen triggered internal therapy. This complementary delivery mechanism matches the dynamic progression of soilborne diseases.

### 2.6 Pathogen‐Responsive Release Mechanism

**FIGURE 6:**
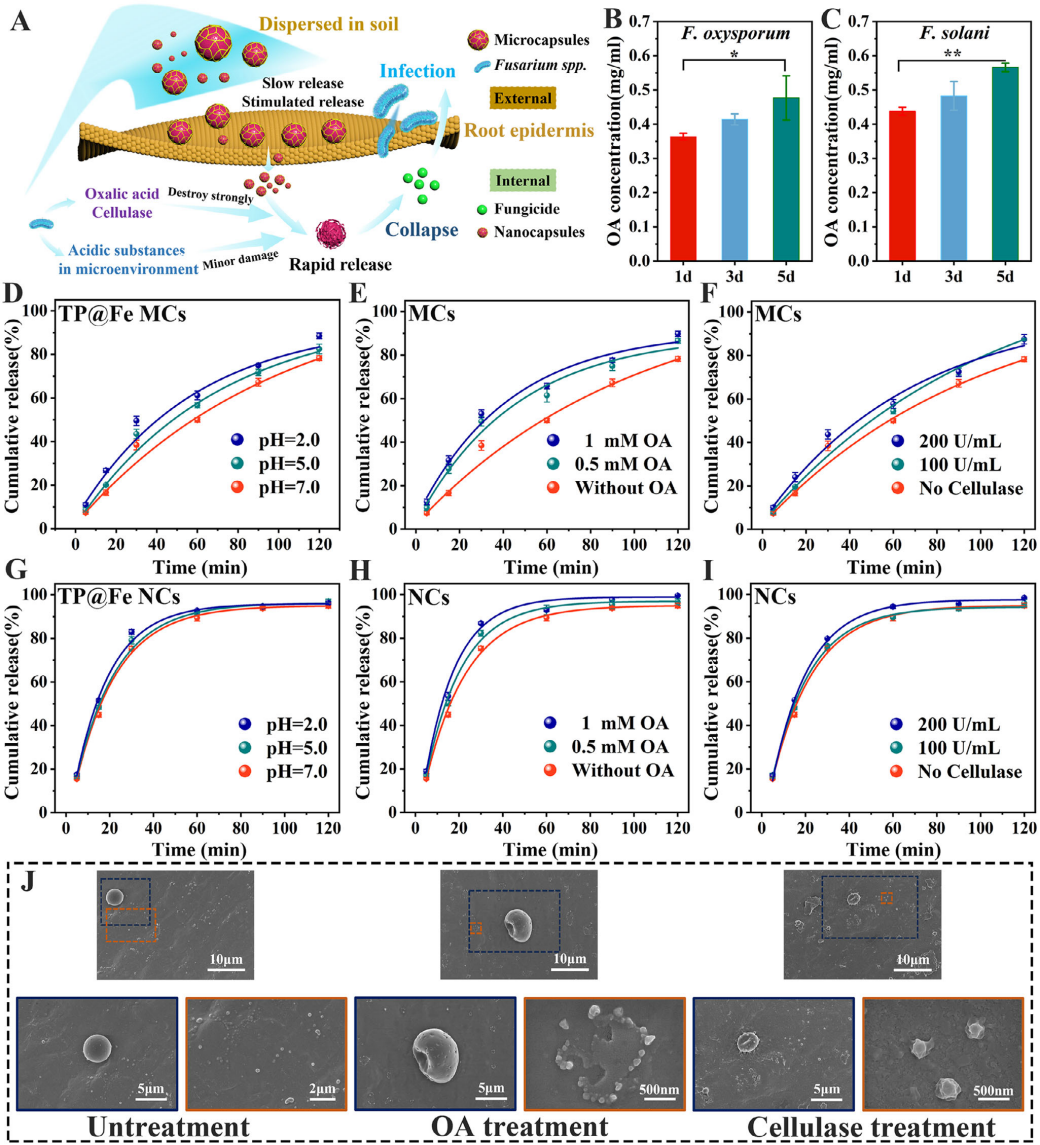
Pathogen‐instructed gating mechanism and responsive release kinetics. (A) Schematic illustration of the spatiotemporal delivery mechanism triggered by fungal virulence factors. (B, C) Quantification of OA secretion profiles during the growth of (B) *F. oxysporum* and (C) *F. solani*. (D‐F) Cumulative release of TP@Fe MCs and (G–I) TP@Fe NCs triggered by acidic pH, OA concentration gradients, and cellulase. (J) SEM images visualizing the structural collapse and disassembly of the carriers upon exposure to the specific triggers.

Achieving effective disease management requires not only precise spatial delivery but also precise temporal activation. Having established the translocation capability of the carriers, we focused on engineering a gating mechanism that responds specifically to fungal infection (Figure 6A). Fungal pathogens modify their microenvironment by secreting virulence factors such as oxalic acid (OA) and hydrolytic enzymes [46, 47, 48, 49]. To validate the physiological relevance of OA as a trigger for responsive release, the secretion profiles of *F. oxysporum* and *F. solani* were quantified (Figure 6B,C). Both pathogens exhibited a consistent, time‐dependent secretion of OA. This accumulation generates a localized acidic and chelating microenvironment that differs from healthy soil conditions [50]. Consequently, we utilized this specific pathological signature to trigger the on‐demand disassembly of the metal‐phenolic network.

To analyze the release kinetics in response to these cues, the NMCs‐88:12 formulation was fractionated into micro‐ and nanoscale populations. We first examined the pH sensitivity. The two populations exhibited distinct release behaviors. The microcapsules displayed a stimuli‐responsive profile characterized by relatively slow release under neutral conditions (pH 7.0) and significantly accelerated rates under acidic conditions (pH 2.0) (Figure 6D, Figure S18). This behavior suggests the function of microcapsules as stable reservoirs capable of responding sensitively to environmental shifts. In contrast, the nanocapsules exhibited a rapid release profile across all pH levels where the cumulative release exceeded 80% within 60 min (Figure 6G). Although acidity slightly enhanced the kinetics, the difference between the treated and control groups was less pronounced compared to the microcapsules. This rapid release is attributed to the high specific surface area and short diffusion pathways of the nanocarriers. While this characteristic implies lower retention stability, it is highly advantageous for curative applications as it ensures that the fungicide becomes immediately bioavailable upon reaching the target internal tissues. Next, the responsiveness to the specific chemical trigger was tested using OA (Figure 6E,H, Figure S19). Both fractions exhibited a dose‐dependent increase in release rates. Increasing the OA concentration from 0 to 1.0 mm caused rapid release. This behavior stems from a synergistic mechanism where OA induces local acidification and, crucially, sequesters the Fe^3+^ centers via competitive chelation, leading to the collapse of the coordination network.

In addition to chemical triggers, enzymatic sensitivity was evaluated (Figure 6F,I, Figure S20). Microcapsules exhibited concentration‐dependent sensitivity to cellulase. In contrast, nanocapsules remained stable at 100 U/mL and required 200 U/mL to show a response. Scanning electron microscopy confirms this trigger‐induced disassembly (Figure 6J). Unlike the intact spherical morphology of untreated controls, capsules exposed to OA or cellulase deformed into amorphous films. Collectively, these results validate a pathogen‐instructed mechanism where the metabolic activity of the fungus, specifically competitive chelation and enzymatic degradation, serves as the molecular switch to drive precise payload release. Importantly, this pathogen‐instructed disassembly can be distinguished from simple environmental soil acidification. General soil acidification induces only limited release. In contrast, the rapid collapse of the TP‐Fe^3^^+^ network is specifically driven by the pathogen's unique combination of oxalic acid chelation and cellulase degradation.

The disassembly of the MPN shell depends on a specific concentration threshold. This allows it to selectively respond to the abnormally high oxalic acid and cellulase levels during *Fusarium* infection, easily distinguishing severe pathogenic attacks from the background activity of beneficial microbes. Moreover, the sequential and trigger‐responsive delivery arising from the distinct properties of micro‐ and nanocapsules establishes the temporal regulation of fungicidal activity.

### 2.7 Environmental Biosafety Evaluation in Zebrafish

**FIGURE 7:**
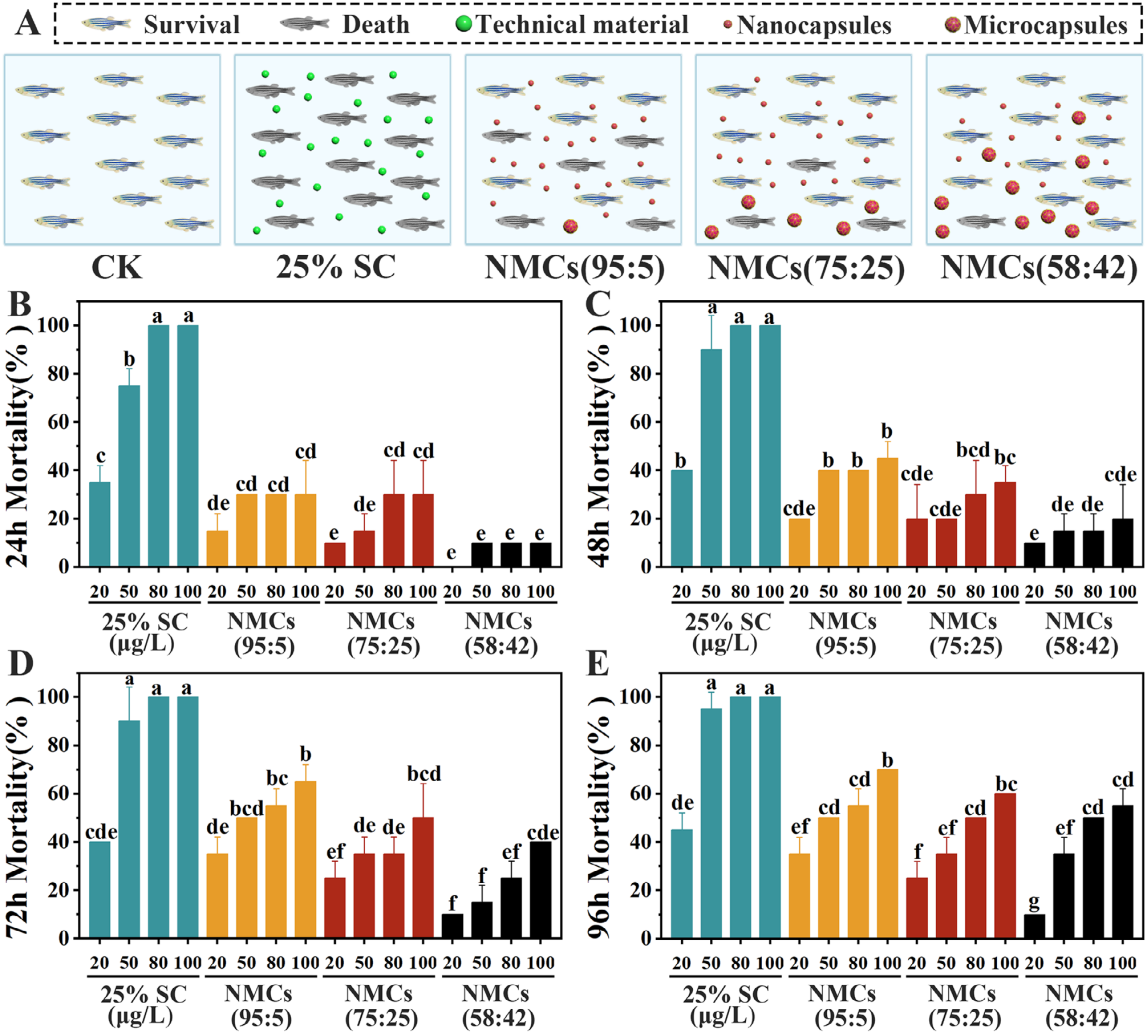
Ecotoxicological assessment and size‐dependent biosafety profile in a zebrafish model. (A) Schematic illustration of the acute toxicity exposure assay comparing the commercial formulation and NMCs. (B–E) Time‐dependent mortality rates of zebrafish exposed to varying concentrations of pyraclostrobin formulations at 24, 48, 72, and 96 h.

Minimizing toxicity toward non‐target organisms is critical for sustainable agriculture. Therefore, the biosafety of the prepared multiscale system was evaluated using zebrafish (*Danio rerio*) as a model. Acute toxicity assays show a difference in mortality rates between the formulations (Figure 7B–E). The commercial 25% SC caused high mortality throughout the 96 h exposure, even at low concentrations. Mortality rates exceeded 70% within the initial 24 h and reached 100% by 72 h at concentrations 50 µg/L. These results indicate the ecological risks associated with the bioavailability of conventional pesticide formulations in aquatic environments [51].

Crucially, supramolecular encapsulation significantly attenuated acute toxicity across all engineered formulations compared to the commercial 25% SC control. This enhanced biosafety arises from the robust metal‐phenolic core–shell architecture which effectively prevents the immediate burst release of the bioactive payload in aquatic environments. A size‐dependent interaction profile was observed where the nano‐dominant NMCs‐95:5 system exhibited slightly elevated mortality relative to micro‐rich variants due to enhanced permeability across biological membranes such as zebrafish gills [52]. Nevertheless, even this most active formulation remained substantially safer than the commercial SC. Our platform transforms the functional divergence between the systemic mobility of nanocarriers and the environmental inertness of microcapsules into an optimization opportunity rather than a rigid functional trade‐off. The precise regulation of the micro‐to‐nano population ratio enables the effective decoupling of therapeutic efficacy from ecological toxicity. This capability allows for the engineering of balanced formulations that retain the high systemic activity required for disease control while maintaining ecotoxicity within an acceptable safety corridor. Consequently, this work presents a versatile safety‐by‐design strategy to reconcile the conflicting demands of high‐potency crop protection and environmental sustainability.

## 3 Conclusion

This study presents a strategy for engineering agricultural materials based on the composition‐dependent liquid‐liquid phase separation of natural polyphenols and non‐ionic surfactants. The interfacial titration strategy employs metal‐ligand coordination to solidify the fluid templates. While the current proof‐of‐concept is validated within the TP‐Fe^3^^+^ system, this supramolecular coacervation and coordination‐solidification mechanism may be extended to other natural polyphenols and metal ions in future studies. This one‐pot protocol allows for the regulation of the microscale and nanoscale proportions. The resulting multiscale architecture achieves a functional division wherein stationary microcapsules serve as reservoirs for preventative protection while nanocapsules act as vectors for systemic curative action. Moreover, the pathogen‐responsive mechanism triggers release in response to fungal virulence factors. This design addresses the trade‐off between therapeutic potency and environmental safety to improve efficacy against root rot. Overall, this work establishes a strategy for designing responsive supramolecular systems through the controlled solidification of fluid interfaces and offers a potential solution for modern crop protection.

## 4 Experimental Section

### 4.1 Materials

Unless otherwise specified, all chemicals were of analytical grade and used as received. Iron(III) chloride hexahydrate (FeCl_3_·6H_2_O), tea polyphenols (TP, Catalog No. T418534, predominantly composed of Epigallocatechin gallate, C_22_H_18_O_11_), fluorescein isothiocyanate (FITC), and cellulase (derived from *Aspergillus niger*) were purchased from Aladdin Biochemical Technology Co., Ltd. (Shanghai, China). The chemical structure of the primary constituent is provided in Figure S21. Technical grade pyraclostrobin (98% purity) was kindly supplied by Hailir Pesticides and Chemicals Group (Qingdao, China). Oxalic acid (OA) was obtained from Tianjin Damao Chemical Reagent Factory and styrene phenol polyoxyethylene ether (SPPE), polyalkoxylated butyl ether (NS‐500LQ), polyoxyethylene castor (EL‐40), alkylphenol polyoxyethylene ether (OP‐10), sorbitan monooleate (Span‐80), polyoxyethylene sorbitan monooleate (Tween‐80) and fatty alcohol polyoxyethylene ether (AEO‐9) were obtained from Haian Petrochemical Plant. Organic solvents, comprising cyclohexanone, n‐hexane, and HPLC‐grade acetonitrile, were procured from Tianjin Kemiou Chemical Reagent Co., Ltd. Deionized water was used for all aqueous preparations.

### 4.2 Preparation of TP@Fe NMCs

The metal‐phenolic capsules were prepared via an interfacial titration strategy. The organic precursor phase consisted of pyraclostrobin and SPPE dissolved in cyclohexanone. Concurrently, the aqueous phase was prepared by dissolving TP in deionized water at specific concentrations. An oil‐in‐water (O/W) emulsion was generated by the gradual introduction of the aqueous phase into the organic phase under continuous mechanical agitation at 800 rpm. This pre‐emulsion was equilibrated for 10 min at ambient temperature to allow interfacial assembly. Subsequently, an aqueous iron(III) chloride solution was added dropwise to initiate metal‐ligand coordination. Following a 30 min stirring period, the system pH was adjusted to 7.0–8.0 using NaOH (1.0 m). The final dispersion was designated as TP@Fe NMCs. The micro‐to‐nano fractional distribution was regulated by varying the mass ratio of TP to SPPE across the precursor phases.

Fluorescently labeled NMCs were synthesized to track translocation pathways. Fluorescein isothiocyanate (FITC) was pre‐dissolved in the organic phase for encapsulation within the capsule core. All subsequent emulsification, coordination, and titration steps remained identical to the protocol for unlabeled NMCs.

### 4.3 Morphological and Structural Characterization

To separate distinct carrier populations, the as‐synthesized dispersion was fractionated via differential centrifugation (5000 rpm, 3 min). Pyraclostrobin content within the separated nanocapsule and microcapsule fractions was quantified by HPLC. Hydrodynamic size distributions were determined via dynamic light scattering (DLS) using a Zetasizer Nano ZSE (Malvern Panalytical) and surface charge properties were assessed using a Zetasizer Lab Blue system. Morphology was visualized by field‐emission scanning electron microscopy (FE‐SEM, Hitachi SU8010) at an accelerating voltage of 5.0 kV. The core–shell structure was observed via transmission electron microscopy (TEM, Thermo Fisher Talos F200X) operating at 200 kV. Surface chemical states were analyzed by x‐ray photoelectron spectroscopy (XPS, Thermo ESCALAB 250Xi) with a monochromatic Al Kα source. Intermolecular interactions were characterized by Fourier‐transform infrared spectroscopy (FTIR, Nicolet 6700). Finally, thermal stability profiles were recorded using thermogravimetric analysis (TGA) from 25 to 600°C under nitrogen flow at a heating rate of 10°C/min.

### 4.4 Thermodynamic and Molecular Weight Analysis

The number‐average (Mn) and weight‐average (Mw) molecular weights of SPPE were determined via gel permeation chromatography (GPC) using a Waters 2695 system equipped with an Ultrahydrogel column and a refractive index detector [53]. Elution was performed using an aqueous 0.1 m NaNO3 mobile phase at a flow rate of 1.0 mL/min with calibration against dextran standards (Table S1). To determine intermolecular association forces, thermodynamic binding parameters were quantified via isothermal titration calorimetry (ITC) on a Nano ITC instrument [54, 55]. The SPPE solution was loaded into the sample cell and titrated against the TP solution at 25°C under constant stirring. The resulting heat profiles were integrated and fitted to an independent binding model using NanoAnalyze software to obtain the enthalpy (ΔH), entropy (ΔS), and Gibbs free energy (ΔG). ΔG is calculated from the fundamental equation: ΔG = ΔH‐TΔS

### 4.5 Density Functional Theory (DFT) Calculations

DFT calculations were performed using the CASTEP module in Materials Studio. The exchange‐correlation interactions were described by the generalized gradient approximation (GGA) with the Perdew‐Burke‐Ernzerhof (PBE) functional, and ultrasoft pseudopotentials were employed. A plane‐wave cut‐off energy of 580 eV was applied to ensure energy convergence. Brillouin zone sampling was conducted using a Monkhorst‐Pack k‐point mesh with a spacing of approximately 0.05 1/Å. The convergence criteria were set to 1.0 × 10^−^^6^ eV/atom for total energy, 0.03 eV/Å for atomic forces, and 1.0 × 10^−^^3^ Å for atomic displacements.

### 4.6 Molecular Dynamics Simulations

All‐atom molecular dynamics (MD) simulations were conducted using the GROMACS 2021.4 package. The OPLS‐AA force field defined the parameters for SPPE, pyraclostrobin, and cyclohexanone whereas the CHARMM general force field (CGenFF) was applied to TP. Water molecules were described by the TIP3P model. Biphasic simulation systems were constructed with a cyclohexanone organic layer and an aqueous phase containing SPPE and TP molecules at experimentally relevant mass ratios. Energy minimization was achieved via the steepest descent algorithm followed by 1 ns equilibration steps under NVT and NPT ensembles. Production trajectories were integrated for 50–100 ns using a 2 fs time step. Temperature was maintained at 298 K via a Nosé‐Hoover thermostat and pressure was coupled to 1 bar using a Parrinello‐Rahman barostat. Periodic boundary conditions were applied in all three dimensions. Trajectory analysis quantified interfacial mass density profiles and radial distribution functions (RDF) to analyze the hydrogen‐bonding network and supramolecular ordering at the oil‐water interface. Molecular visualizations were generated using VMD software. To ensure computational reproducibility, C_22_H_18_O_11_ was selected as the specific representative molecular model for the tea polyphenol mixture. For the non‐ionic surfactant SPPE (POE_16_), the molecular model employed in simulations retained the polyoxyethylene hydrophilic chains alongside the aromatic and alkyl hydrophobic motifs, capturing its representative amphiphilic architecture. The exact molecular topologies and 3D configurations employed in these MD simulations are presented in Figure S8.

### 4.7 Encapsulation Efficiency and Release Profiles

Encapsulation efficiency and loading capacity were assessed by quantifying non‐encapsulated pyraclostrobin within the supernatant via HPLC after centrifugation. In vitro release profiles for both bulk NMCs and fractionated subpopulations were evaluated using the dialysis bag technique. Experiments were conducted under different pH conditions (2.0, 5.0, 7.0) and in the presence of biological triggers such as OA and cellulase. Chromatographic conditions and calculation protocols are described in the Supporting Information.

### 4.8 Fungal Metabolite Secretion Assay

To determine the pathogen‐specific triggers, the metabolic secretion profiles of *F. solani* and *F. oxysporum* were characterized. Standardized mycelial discs (5 mm) were inoculated into sodium carboxymethylcellulose liquid medium and incubated at 28°C under constant orbital shaking. Cell‐free culture supernatants were collected via centrifugation at specific time points (1, 3, and 5 days post‐inoculation). The concentration of secreted OA was quantified spectrophotometrically using a commercial colorimetric assay kit following the manufacturer's protocol.

### 4.9 In Vitro Antifungal Activity

The antifungal activity was evaluated using the mycelial growth inhibition method. Potato dextrose agar plates were supplemented with the NMCs formulations or commercial controls at serial concentrations. A standardized mycelial plug (5 mm) taken from the edge of an actively growing colony was placed centrally onto the treated medium. The plates were incubated at 25°C in the dark. Colony diameters were measured using the cross‐line method after 6 days of incubation. The percentage of growth inhibition was calculated relative to the untreated control and the half‐maximal effective concentration (EC_50_) was calculated via regression analysis.

### 4.10 Pot Experiments

Efficacy against peanut root rot was evaluated in a controlled greenhouse setting. For the curative treatment, pre‐germinated peanut seeds Yuhua 22 were sown into soil artificially inoculated with *F. oxysporum* using colonized millet seeds [56]. Seedlings were grown for 7 days to allow infection establishment, after which treatments comprising the commercial suspension concentrate and various encapsulated formulations were applied via root drenching. For the protective treatment, pre‐germinated seeds were sown in healthy soil. At 7 days post‐germination, seedlings were treated with the formulations via root drenching, followed by artificial inoculation with *F. oxysporum* 48 h later.

Disease severity was quantified 15 days post‐treatment or post‐inoculation using a six‐point grading scale. A score of 0 indicates healthy plants with no observable lesions. Intermediate scores represent progressive infection severity: score 1 indicates scattered lesions on the stem base; score 2 involves lesions covering less than 25% of the root area; score 3 corresponds to 25–50% coverage; and score 4 indicates 50%–75% coverage. Finally, a score of 5 represents complete necrosis of the stem base and root system.

### 4.11 Translocation and Distribution

Systemic mobility was assessed using peanut plants cultivated in a hydroponic setup to avoid soil interference. The root systems were immersed in a suspension containing FITC‐labeled NMCs at a concentration of 100 mg/L. Following a 48 h uptake period, root and stem tissues were harvested and rinsed thoroughly with deionized water to remove surface residues. Thin transverse sections were prepared manually and mounted for microscopy. The spatial distribution of the carriers was observed via confocal laser scanning microscopy using a Leica TCS SP8 system. Excitation was set at 488 nm with emission collection in the 500–550 nm range. Finally, vascular localization was verified by merging the fluorescence signals with bright‐field transmission channels.

### 4.12 Biosafety Assessment

Acute toxicity evaluations were conducted using adult zebrafish (*Danio rerio*) according to OECD Guideline 203. Prior to exposure, fish populations underwent a 7‐day acclimatization period under controlled laboratory conditions maintained at 26 ± 1°C and a 12 h light/12 h dark photoperiod. Zebrafish were randomly distributed into test tanks with 20 fish per group and exposed to different concentrations of either the commercial suspension concentrate or the engineered NMCs. A control group was maintained simultaneously in aerated dechlorinated tap water without chemical additives. Mortality rates were recorded at 24 h intervals throughout the 96 h exposure window. Death was confirmed by the complete cessation of respiratory movement and the lack of response to mechanical stimuli.

### 4.13 Statistical Analysis

Data analysis was performed using SPSS 26.0 software. All quantitative results are presented as the mean ± standard error (SE) calculated from at least three independent replicates. Statistical significance was evaluated using one‐way analysis of variance followed by Tukey's post–hoc test. Differences between two specific groups were compared using an independent samples *t*‐test. Differences were considered statistically significant at ^*^ *p* < 0.05 and highly significant at ^**^ *p* < 0.01. Different lowercase letters indicate significant differences among groups (*p* < 0.05).

## Author Contributions

T. J. and L. Z. conceived the project and designed the experiments. R. Z. performed the experiments. L. C., L. H., K. C., Y. S., X. M. supervised the experimental work. T. J. and R. Z. wrote the paper with input from all authors.

## Conflicts of Interest

The authors declare no conflicts of interest.

## Supporting information




**Supporting File 1**: advs77149‐sup‐0002‐SuppMat.docx.

**Supporting File 2**: advs77149‐sup‐0001‐Movie1.mp4.

## Data Availability

The data that support the findings of this study are available from the corresponding author upon reasonable request.
