# Supplementary material for: Programmed Interfacial Phase Separation for Spatiotemporal Fungicide Delivery via Tunable Micro/Nano Architectures
Source: Adv Sci (Weinh). 2026 Aug 11;13(61):e77149. doi: 10.1002/advs.77149 (PMC13460277; doi:10.1002/advs.77149)
Supplement: Supplementary file 1 [file ADVS-13-e77149-s001.docx]

**Programmed Interfacial Phase Separation for Spatiotemporal Fungicide Delivery via Tunable Micro/Nano Architectures**

*Rui Zhang ^a,b,c^, Tongfang Jing ^a,b,c*^， Leiming He ^a,b,c^, Kaidi Cui ^a,b,c^, Yuying Song ^a,b,c^, Xuewei Mao ^a,b,c^, Lidong Cao ^d *^, Lin Zhou ^a,b,c *^*

^a^ College of Plant Protection, Henan Agricultural University, Zhengzhou, Henan, 450046, P. R. China.

^b^ Henan Key Laboratory of Creation and Application of New Pesticide, Henan Agricultural University, Zhengzhou, Henan, 450046, P. R. China.

^c^ State Key Laboratory of High-Efficiency Production of Wheat-Maize Double Cropping, Zhengzhou, Henan, 450046, P. R. China.

^d^ Institute of Plant Protection, Chinese Academy of Agricultural Sciences, Beijing, 100193, P. R. China.

Corresponding authors

Tongfang Jing*, Email: 18706381835@163.com

Lidong Cao*, Email: caolidong@caas.cn

Lin Zhou*, Email: zhoulinhenau@163.com

**HPLC Analysis**

High-Performance Liquid Chromatography (HPLC) analysis was performed using an Agilent 1200 system (Agilent Technologies, USA) equipped with a UV-Vis detector. Separation was achieved on a C18 reverse-phase column (Agilent Zorbax SB-C18, 4.6 mm × 250 mm, 5 μm particle size). The mobile phase consisted of acetonitrile and water (80:20, v/v) at a flow rate of 1.0 mL/min. The column temperature was maintained at 25 °C. The detection wavelength was set at 275 nm. The injection volume was 10 μL. A calibration curve was established using standard solutions of pyraclostrobin (6.25-100 mg/L) in methanol.

**Encapsulation Efficiency and *in Vitro* Release Studies**

**Encapsulation efficiency (EE)**

The TP@Fe NMCs suspension (0.50 g) was weighed into a clean, dry glass bottle (100 mL), and 40 mL of deionised water was added. Subsequently, 40 mL of *n*-hexane was added to the glass bottle, which was quickly placed on a roller mixer (MT-T6-Pro, SCILOGEX) at 70 rpm for 5 min, and the upper *n*-hexane layer (0.5 mL) containing unencapsulated pyraclostrobin (*M*_t_) was promptly extracted for measurement. And then, the TP@Fe NMCs suspension (0.50 g) was weighed into a clean, dry volumetric flask (50 mL) again, and then about 40 mL of methanol was added to the volumetric flask. The volumetric flask was ultrasonically shaken and extracted for 30 min. and then use the methanol to a constant volume of 40 mL and shaken well it. The upper methanol layer (0.5 mL) was extracted for measurement. Calculated all amounts of pyraclostrobin (M_0_) in 0.50 g TP@Fe NMCs suspension from the measured concentration. All measurements were performed in triplicate (n=3).

The EE was then calculated using the following equation:

EE (%) = [(m_0_ - m_t_) / m_0_] × 100%.

***In Vitro* Release Behavior**

The release profiles of TP@Fe NMCs were measured according to previous reports, with minor modifications^[1]^. First, aqueous suspensions of the NMCs were first diluted with deionized water to achieve a pyraclostrobin concentration equivalent to 100.0 mg/L. For each release study, a 40.0 mL aliquot of this diluted NMCs suspension was carefully transferred into a clean glass bottle equipped with an airtight seal. Subsequently, 40.0 mL of *n*-hexane was added to the bottle, establishing a biphasic system. The NMCs remained dispersed in the initial 40.0 mL aqueous phase, and the released pyraclostrobin partitioned into the overlaying *n*-hexane phase. The sealed bottles were then incubated at a controlled temperature of 25.0 ± 0.5 °C and agitated on a roller mixer at a constant speed of 70 rpm to facilitate uniform mixing and mass transfer. At different time points, the upper n-hexane layer (0.5 mL) was removed and quickly added to an equal volume of n-hexane. The cumulative release proportions of pyraclostrobin from the TP@Fe NMCs were calculated using the following equation:

Cumulative release proportion (%) = [(C_t_×V_0_-M_t_)/M_a_] ×100%

Where *C*_t_ is the pyraclostrobin concentration, *V*_0_ is the volume of *n*-hexane, and *M*_a_ are the total amounts of pyraclostrobin.

**Figure S1.** The influence of varying concentrations of tea polyphenols in combination with seven representative non-ionic surfactants (Amphiphilic molecules) on the extent of phase separation in aqueous phase.

**Figure S2.** Differences in the degree of phase separation of emulsions formed by five representative non-ionic surfactants (Amphiphilic molecules) in the presence of varying concentrations of tea polyphenols.

**Figure S3.** After the emulsion phase separation, the upper and lower liquid layers have fluidity at different angles.

**Table S1.** Molecular weight results determined using gel permeation chromatography (GPC).

| Sample | Retention time | Mn | Mw | MP | Area | Area (%) |
| --- | --- | --- | --- | --- | --- | --- |
| SPPE | 18.965 min | 944 | 2963 | 1793 | 1028832 | 100% |

**Table S2.** The influence of different content components on the NCs/MCs ratio.

| TP (%) | SPPE (%) | NCs/MCs |
| --- | --- | --- |
| 1 | 5 | 95:5 |
| 1.5 | 6 | 88:12 |
| 1.5 | 5 | 75:25 |
| 1.5 | 4 | 65:35 |
| 2 | 5 | 58:42 |

**Figure S4**. SEM images of the dried samples obtained from the upper supernatant phase and lower coacervate phase after 12 h standing-induced phase separation of the oil-water biphasic emulsion mixtures containing TP and the non-ionic surfactants NS-500LQ, Tween-80, and EL-40.

**Figure S5**. SEM images of the dried samples obtained from the upper supernatant phase and lower coacervate phase after 12 h standing-induced phase separation of the oil-water biphasic emulsion mixtures containing TP and different contents of SPPE (4%, 5%, and 6%) upon the introduction of Fe^3+^ (acidic conditions), as well as SEM images of the dried samples from the macroscopically homogeneous single-phase system formed after OH⁻-triggered pH modulation.

**Figure S6**. TEM images of the dried samples obtained from the upper supernatant phase (A) and lower coacervate phase (B) after 12 h standing-induced phase separation of the oil-water biphasic emulsion mixture containing TP and SPPE upon the introduction of Fe³⁺ (acidic conditions), as well as TEM images of the dried samples from the macroscopically homogeneous single-phase system formed after OH⁻-triggered pH modulation (C).

**Figure S7**. XPS characterization of TP@Fe NMCs. High-resolution C1s (A) and O1s (B) spectra of TP@Fe NMCs.

**Figure S8.** The molecular structure formula used in the MD simulation.

**Figure S9.** Snapshots at different time points illustrating the distribution of molecules from the oil and aqueous phases (5% SPPE–1% tea polyphenol) at the oil/water interface.

**Figure S10.** Snapshots at different time points illustrating the distribution of molecules from the oil and aqueous phases (5% SPPE–2% tea polyphenol) at the oil/water interface.

**Figure S11.** Density distributions along the Z-axis of tea polyphenols, pyraclostrobin and surfactant in the two systems during the 80-100 ns time interval. (A) 5% SPPE–1% tea polyphenol. (B) 5% SPPE–2% tea polyphenol.

**Figure S12.** DTG curves assessing thermal stability of pyraclostrobin, TP@Fe NCs and TP@Fe MCs.

**Figure S13.** The cumulative release kinetics curves of TP@Fe NMCs formed by SPPE and varying concentrations of tea polyphenols were fitted using the zero-order mathematical model (A), First-order mathematical model (B), Higuchi mathematical model (C) and Ritger-Peppas mathematical model (D).

**Figure S14.** The cumulative release kinetics curves of TP@Fe NMCs formed by tea polyphenols and varying concentrations of SPPE were fitted using the zero-order mathematical model (A), First-order mathematical model (B), Higuchi mathematical model (C) and Ritger-Peppas mathematical model (D).

**Figure S15**. (A, B) Infection images of inoculated plants randomly selected from three replicate groups before fungicide application (A), and the relative biomass of *F. oxysporum* in the infected tissues (B).

**Figure S16**. Relative *F. oxysporum* biomass in peanut tissues from different treatment groups at 15 days after curative treatment.

**Table S3.** Primers used for sequencing and qPCR in this study.

| **Function** | **Primers Name** | **Sequence** | **Application style** | **Reference** |
| --- | --- | --- | --- | --- |
| Ahactin 7 | F | GATTGGAATGGAAGCTGCTG | qPCR | [2] |
|  | R | CGGTCAGCAATACCAGGGAA |  |  |
| *F. oxysporum* | ITS1F | CTTGGTCATTTAGAGGAAGTAA | qPCR | [3] |
|  | AFP308R | CGAATTAACGCGAGTCCCAA |  |  |

**Figure S17.** CLSM images showing systemic distribution of fluorescently-labeled NMCs in stems on the second day.

**Figure S18.** The cumulative release kinetics curves of TP@Fe MCs under different pH conditions were fitted using the zero-order mathematical model (A), First-order mathematical model (B), Higuchi mathematical model (C) and Ritger-Peppas mathematical model (D).

**Figure S19.** The cumulative release kinetics curves of TP@Fe MCs under different oxalic acid concentrations were fitted using the zero-order mathematical model (A), First-order mathematical model (B), Higuchi mathematical model (C) and Ritger-Peppas mathematical model (D).

**Figure S20.** The cumulative release kinetics curves of TP@Fe MCs under different cellulase concentrations (U/mL) were fitted using the zero-order mathematical model (A), First-order mathematical model (B), Higuchi mathematical model (C) and Ritger-Peppas mathematical model (D).

**Figure S21**. Schematic diagram of the chemical structure of tea polyphenols.

**Movie S1.** Phase Separation of an Emulsion Formed Between a Tea Polyphenol-Containing Aqueous Phase (1% and 2% TP) and a Non-Ionic Surfactant-Containing Oil Phase (5% SPPE).

**References**

[1]	R. Wang, F. Luan, Z. Wang, X. Liu, X. Zhang, K. Wang, W. Mu, F. Liu, D. Zhang, Spontaneously formed ratio-tunable micro- and nano-capsule coexist system for precision and on-demand fungicide delivery in crop leave, *Adv. Mater.* **36** (2024) 1–15.

[2]	H. Xue, K. Zhao, X. Dong, Q. Ma, S. Hu, Z. Cao, Y. Shu, Y. Li, X. Huang, K. Zhao, D. Qiu, W. Shao, R. Ren, Z. Li, F. Gong, X. Ma, C. Wei, D. Yin, PeanutOmics: A comprehensive platform with an integrative multi-omics atlas for peanut research, *Plant Commun.* **7** (2026) 101622.

[3]	G. Lei, Z. Han, X. Wang, A. Malacrinò, T. Kang, D. Zhang, J. Zhang, Z. Zhang, H. Wu, Synthetic microbial communities rescues strawberry from soil-borne disease by enhancing soil functional microbial abundance and multifunctionality, *J. Adv. Res.* **83** (2026) 113–128.
